# Factors Influencing Poststroke Cognitive Dysfunction: Cross-Sectional Analysis

**DOI:** 10.2196/59572

**Published:** 2024-11-19

**Authors:** Wu Zhou, HaiXia Feng, Hua Tao, Hui Sun, TianTian Zhang, QingXia Wang, Li Zhang

**Affiliations:** 1 School of Medicine Southeast University Nanjing China; 2 Zhongda Hospital Southeast University Nanjing China; 3 Huai 'an Second People's Hospital Huaian China; 4 The First People's Hospital of Hefei HeFei China

**Keywords:** stroke, cognitive dysfunction, analysis of associated factors, MMSE, Mini-Mental State Examination, status survey, cognitive, survey, cognitive impairment, cross-sectional study, cross sectional, stroke patients, cognition, education

## Abstract

**Background:**

Poststroke cognitive impairment (PSCI) is a common and debilitating complication that affects stroke survivors, impacting memory, attention, and executive function. Despite its prevalence, the factors contributing to PSCI remain unclear, with limited insights into how demographic and clinical variables influence cognitive outcomes.

**Objective:**

This study investigates the incidence of cognitive impairment in patients with stroke and examines key demographic and clinical factors, such as age, gender, and education level, which contribute to cognitive decline. The aim is to provide a deeper understanding of PSCI to inform early intervention strategies for improving patient outcomes.

**Methods:**

A cross-sectional study was conducted on 305 patients with ischemic stroke admitted to Zhongda Hospital, Southeast University, from January 2019 to September 2022. Cognitive function was assessed using the Mini-Mental State Examination (MMSE) within 72 hours of hospital admission. Demographic information, including age, gender, and education level, were collected. Statistical analyses were performed using chi-square tests, independent *t* tests, and multivariate regression to assess the relationship between cognitive function and key variables. Pearson correlation analysis explored associations among age, education, and MMSE scores.

**Results:**

Among the 305 patients with stroke, 16.7% (n=51) were diagnosed with cognitive impairment based on MMSE scores. The prevalence of cognitive impairment was slightly higher in males (17.6%, n=159) than females (15.8%, n=146), but this difference was not statistically significant. A strong negative correlation was found between MMSE scores and age (*r*=–0.32; *P*<.01), indicating that older patients had lower cognitive function. Education level showed a positive correlation with MMSE scores (*r*=0.41; *P*<.01), with patients with higher educational attainment demonstrating better cognitive outcomes. Cognitive function showed a marked decline in patients older than 60 years, particularly in domains such as memory, attention, and language skills.

**Conclusions:**

This study confirms that age and education are significant factors in determining cognitive outcomes after stroke. The results align with existing literature showing that cognitive function declines with age, while higher educational attainment serves as a protective factor. The findings suggest that individuals with greater cognitive reserve, often linked to higher education, are better equipped to cope with the impact of brain injury. However, the study’s reliance on MMSE may have limited its ability to detect domain-specific impairments. Future studies should consider using more sensitive cognitive tools, such as the Montreal Cognitive Assessment (MoCA), to provide a more comprehensive evaluation of PSCI. Cognitive impairment is prevalent among stroke survivors, with age and education level being key factors influencing outcomes. These findings underscore the importance of early detection and targeted interventions to mitigate cognitive decline. Further research with larger samples and more sensitive cognitive assessments is needed to fully understand PSCI and improve rehabilitation strategies for patients with stroke.

## Introduction

Stroke is a neurological condition characterized by sudden damage to brain tissue resulting from disrupted cerebral blood flow due to various causes, leading to a range of clinical symptoms that persist for more than 24 hours or result in death [1]. Stroke is associated with high morbidity, disability, recurrence, and mortality rates. In China, stroke remains the leading cause of death [2]. Globally, ischemic strokes account for 75% to 90% of all stroke cases, while hemorrhagic strokes comprise 10% to 25% [3,4]. The incidence of stroke is on the rise, with approximately 15 million new cases annually worldwide and about 1.5 to 2 million new cases each year in China alone. Notably, the stroke incidence rate in China is increasing by 9% per year. Among stroke survivors, 70% to 80% experience varying degrees of functional impairment, with cognitive dysfunction being one of the most significant and disabling consequences [5].

Poststroke cognitive impairment (PSCI) is a major complication following a stroke, encompassing a spectrum from mild cognitive impairment (without dementia) to poststroke dementia. PSCI can emerge early, even in the hyperacute stage of stroke, characterized by deficits in memory, attention, executive function, and language abilities. These deficits severely impact patients’ daily living activities, social participation, and overall quality of life [6,7]. Epidemiological studies indicate that cognitive dysfunction affects approximately 61% of patients with stroke within 10 years post stroke [8], and up to 80% of survivors exhibit some form of cognitive impairment within the first few months following the event [9]. A retrospective study in China reported that 23.35% of patients with acute ischemic stroke experienced cognitive dysfunction within 3 months [10]. Despite the high prevalence, the precise mechanisms underlying PSCI are not fully understood, which complicates efforts to predict outcomes and implement effective interventions. The significant burden of PSCI not only affects patients and their families but also places substantial strain on health care systems and society due to the long-term care needs and associated loss of productivity.

While numerous studies have explored various risk factors for PSCI, such as age, education level, and preexisting health conditions, the specific influences of these factors on cognitive outcomes remain poorly understood. This research has identified age and lower educational attainment as significant risk factors, but the interplay between these factors and the severity and characteristics of the stroke (eg, hemispheric location and extent of brain injury) remains inadequately explored [11]. Furthermore, existing literature has often overlooked the variability in stroke severity, a crucial determinant of cognitive outcomes, and how this interacts with demographic factors to influence the risk and extent of cognitive impairment. This gap highlights the need for a more nuanced understanding of the multifactorial contributions to PSCI.

This study offers a novel perspective by systematically investigating the incidence of cognitive impairment among patients with stroke and examining the associated risk factors, including age, gender, education level, and specific stroke characteristics, within a well-defined cohort. Unlike previous research, our study uses a unique methodological approach that combines both cross-sectional data and sophisticated multivariate analysis techniques to explore these relationships more comprehensively. Moreover, our study includes an analysis of specific cognitive domains affected by stroke, rather than relying solely on global cognitive measures, providing deeper insights into the differential impact of demographic and clinical factors on various aspects of cognitive function.

Additionally, this study explores how demographic factors, such as age and education level, interact with stroke characteristics to influence cognitive outcomes. By examining these interactions, our research provides a more nuanced understanding of the factors contributing to PSCI. The insights gained from this study could help in developing targeted early intervention strategies aimed at reducing the risk of cognitive decline following a stroke, ultimately improving patient outcomes and enhancing clinical practice in stroke rehabilitation.

## Methods

### Study Design and Participants

This cross-sectional study was conducted with patients with stroke admitted to the Department of Neurology at Zhongda Hospital, Southeast University, between January 2019 and September 2022. A total of 307 patients were initially considered for inclusion in the study. However, 2 patients were excluded for being younger than 18 years of age, resulting in a final sample size of 305 patients (159 males and 146 females). Patients were prospectively recruited upon admission to ensure a representative sample of the stroke population. Inclusion criteria are (1) patients diagnosed with ischemic stroke according to the diagnostic criteria established by the Chinese Medical Association and (2) confirmation of ischemic stroke through imaging studies, such as computed tomography (CT) or magnetic resonance imaging (MRI). Exclusion criteria are (1) patients with severe comorbidities, including cardiac, hepatic, or renal organ insufficiency; (2) individuals with coagulation dysfunction; (3) patients with significant respiratory diseases; (4) individuals exhibiting poor compliance or inability to cooperate with study procedures; (5) patients diagnosed with cerebral hemorrhage, those in the acute stage of cerebral infarction, or those with severe cerebral infarction were excluded (the exclusion of patients with severe strokes was necessary because their distinct clinical profiles, including more extensive neurological damage and impaired consciousness, could significantly confound the study outcomes—these patients typically require different clinical management and may have more severe cognitive impairments, which could skew the results and make it difficult to assess the impact of less severe strokes on cognitive function); (6) patients with documented cognitive impairment prior to the stroke event, based on their previous medical history; and (7) patients younger than 18 years of age.

It is important to note that the inclusion criteria did not specify the stage of stroke (acute, subacute, or chronic). This limitation should be considered when interpreting the findings, as the stroke stage could influence cognitive outcomes. Future studies should aim to include patients at different stroke stages to provide more nuanced insights into the progression and impact of PSCI.

The reporting of this research will strictly adhere to the STROBE (Strengthening the Reporting of Observational Studies in Epidemiology) statement, which is provided in Multimedia Appendix 1.

### Data Collection Procedures

#### General Information Survey

Upon admission, general demographic and clinical information, including age, gender, and education level (years of education), was collected prospectively using a standardized questionnaire. For patients who were unable to complete the questionnaire due to their medical condition (eg, aphasia or severe cognitive impairment), a legally authorized representative or caregiver provided the required information to ensure data completeness and accuracy.

#### Assessment of PSCI

Cognitive status was assessed using the Mini-Mental State Examination (MMSE), a widely used and validated tool for detecting cognitive impairment. The MMSE is particularly suitable for its practicality and ease of administration, providing a quick, general overview of cognitive function across several domains. While the MMSE is not specifically tailored to detect vascular cognitive impairment, its use remains widespread in clinical practice due to its applicability in various settings, including individuals with subacute or chronic stroke [12]. Importantly, patients in the acute phase of stroke were excluded from this study, and the cognitive assessments were conducted in individuals beyond the acute stage, further supporting the use of the MMSE, which is often used in post-acute care settings. This aligns with evidence suggesting that the MMSE is an effective tool for monitoring cognitive function in the subacute and chronic phases of stroke recovery [13].

The MMSE was administered within 72 hours of hospital admission by 2 trained specialist nurses to standardize the timing of data collection and minimize variability related to stroke progression or recovery. The MMSE evaluates 5 cognitive domains—orientation, memory, attention and calculation, recall ability, and language skills, with a maximum score of 30 points. Higher scores indicate better cognitive function. The diagnostic criteria for cognitive impairment were adjusted based on educational levels, following established guidelines that account for the influence of education on MMSE performance [14]. Specifically, patients with no formal education were classified as cognitively impaired if their score was below 17 points; patients with up to 6 years of education (elementary level) were considered impaired with scores below 20 points; and those with more than 6 years of education (secondary level or higher), cognitive impairment was defined as scores below 24 points. These thresholds align with recent literature recommendations for the use of MMSE in diverse populations [15].

### Group Analysis and Statistical Methods

The study included multiple group analyses to explore potential differences in cognitive impairment across various demographic factors, such as age, gender, and education level. Gender-based comparisons were made to examine whether cognitive outcomes differed between male and female patients with stroke, given the existing literature suggesting potential gender differences in stroke outcomes and cognitive decline. Additionally, comparisons were made across different age groups and education levels to identify any patterns or associations between these demographic factors and cognitive impairment. These groupings were introduced based on preliminary findings in the literature and were aimed at understanding the influence of these variables on cognitive outcomes.

Data analysis was conducted using the SPSS (version 26.0; IBM Corp) statistical software package. Categorical variables were summarized as frequencies and percentages, with comparisons between groups assessed using the chi-square test. Continuous variables were tested for normality. Normally distributed data were presented as mean (SD) and analyzed using an independent sample *t* test for comparisons between 2 groups. Abnormally distributed data were summarized as medians and IQR and compared using the Mann-Whitney *U* test.

For comparisons involving more than 2 groups, 1-way ANOVA with post hoc analysis was used to identify significant differences in cognitive function across groups based on variables such as age and education. A multivariable analysis was performed using analysis of covariance (ANCOVA) to adjust for potential confounding factors such as age, gender, and education level, ensuring that the effects of these variables were accounted for when assessing group differences in MMSE scores. This method allowed for the isolation of the effects of the primary independent variables while controlling for demographic factors known to influence cognitive function.

Additionally, Pearson correlation analysis was used to assess relationships between continuous variables, such as age and MMSE scores, and years of education and MMSE scores. A *P* value of less than .05 was considered statistically significant.

### Ethical Considerations

This study was conducted in strict accordance with ethical guidelines for research involving human participants. Ethics approval was obtained from the Independent Ethics Committee for Clinical Research of Zhongda Hospital, affiliated with Southeast University (2020ZDKYSB216). Informed consent was obtained from all participants or their legal representatives before data collection. Data confidentiality and participant privacy were strictly maintained, with all data fully anonymized prior to analysis. No financial compensation was provided to participants, as the study involved voluntary participation and prospective data collection. Additionally, no identifiable images or personal data were included in any resulting publications to protect participant confidentiality.

### Study Setting and Recruitment Process

This study was conducted at Zhongda Hospital, Southeast University, a large tertiary medical center that specializes in stroke care, providing services to both urban and rural populations. The hospital’s neurology department is equipped with advanced diagnostic tools such as CT and MRI, ensuring timely and accurate diagnosis of patients with stroke.

Patients were recruited from the neurology ward after a stroke diagnosis was confirmed by imaging. The recruitment process was carried out continuously between January 2019 and September 2022. Eligible patients were identified by the attending neurologists and stroke specialists within 48 hours of admission. Potential participants were approached by the research team, who provided a thorough explanation of the study’s objectives, methods, and confidentiality measures. The inclusion and exclusion criteria were reviewed, and informed consent was obtained from either the patient or, in cases of cognitive or communicative impairments, their legally authorized representatives.

The recruitment process ensured that no patient who met the criteria was missed, and all patients were given the opportunity to participate regardless of their demographic background or stroke severity.

## Result

### Participant Recruitment and Final Sample Size

**Figure 1:**
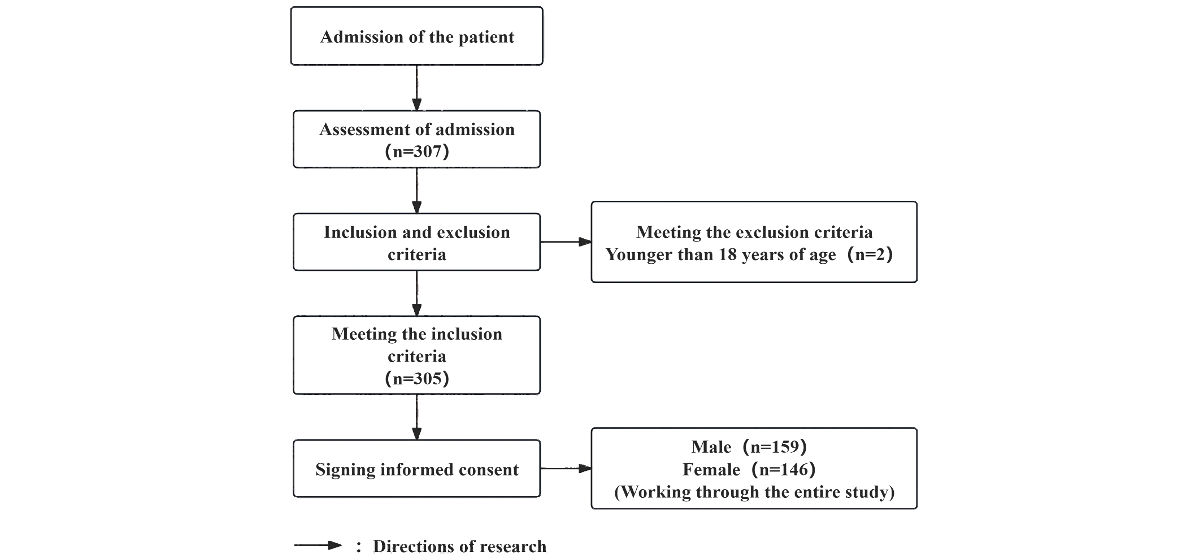
Flowchart of participants included in the study.

A total of 307 patients with stroke were initially recruited for this study. Following the application of the inclusion and exclusion criteria, 2 patients were excluded for being younger than 18 years of age. This resulted in a final sample size of 305 patients, comprising 159 males and 146 females (Figure 1). The exclusion criteria also ensured that patients with severe comorbidities or preexisting cognitive impairment were not included, thereby providing a more homogeneous sample focused on assessing PSCI.

### Prevalence of Cognitive Impairment in Patients With Stroke

Among the 305 patients with stroke included in the study, 51 (16.7%) were found to have cognitive impairment, as determined by their MMSE scores. The prevalence of cognitive impairment was slightly higher in male patients (17.6%, n=159) than in female patients (15.8%, n=146). These findings suggest a relatively comparable prevalence of cognitive impairment across genders within this cohort, with no significant gender-based differences observed in the rate of cognitive impairment.

### Cognitive Status of Patients With Stroke

The results show that no significant differences in cognitive function were observed between male and female patients across various domains, including memory, attention, and language skills. Both genders scored similarly in these areas, with male patients having a median age of 65 (IQR 53-75.25) years compared to 61 (IQR 51-73) years for females; however, this age difference was not statistically significant (*z* score=–1.826; *P*=.07; Table 1). In contrast, it presents results from a multivariate regression analysis, revealing that age is a significant risk factor for cognitive impairment, with a coefficient of –0.050 (95% CI –0.082 to –0.018; *P*=.002), indicating a decline in cognitive scores with each additional year of age. Furthermore, years of education showed a positive association with cognitive outcomes (coefficient=0.212, 95% CI 0.078-0.347; *P*=.002), suggesting that higher educational attainment serves as a protective factor against cognitive decline. Gender did not show a significant effect on cognitive impairment (B=–0.034; *P*=.95). These findings underscore the critical influence of age and education on cognitive impairment in patients with stroke while indicating that gender does not significantly impact cognitive status (Table 2).

**Table 1:** Comparison of cognitive function of patients with stroke by gender.

	Male, median (IQR)		Female, median (IQR)		*z* score		*P* value
Age	65 (53-75.25)		61 (51-73)		–1.826		.07
Directional force	10 (8-10)	10 (8.5-10)		–0.227		.82	
Memory	3 (3-3)	3 (3-3)		–0.732		.46	
Attention and calculation	5 (4-5)	5 (3-5)		–0.295		.77	
Recollection ability	2 (1-3)	2 (1-3)		–0.024		.98	
Language skills	9 (8-9)	9 (8-9)		–0.702		.48	
Total score	28 (25-30)	28 (25-29)		–0.014		.99	

**Table 2:** Multivariate regression analysis of influencing factors of cognitive impairment.

	B (95% CI)	SE	*t* test (*df*)	*P* value
Constant	26.919 (23.663 to 30.175)	1.655	16.270 (304)	.000
Age	–0.050 (–0.082 to –0.018)	0.016	–3.079 (304)	.002
Gender	–0.034 (–1.128 to 1.060)	0.556	–0.061 (304)	.95
Years of education	0.212 (0.078 to 0.347)	0.068	3.106 (304)	.002

### Correlation Between Clinical Variables and Cognitive Function

**Figure 2:**
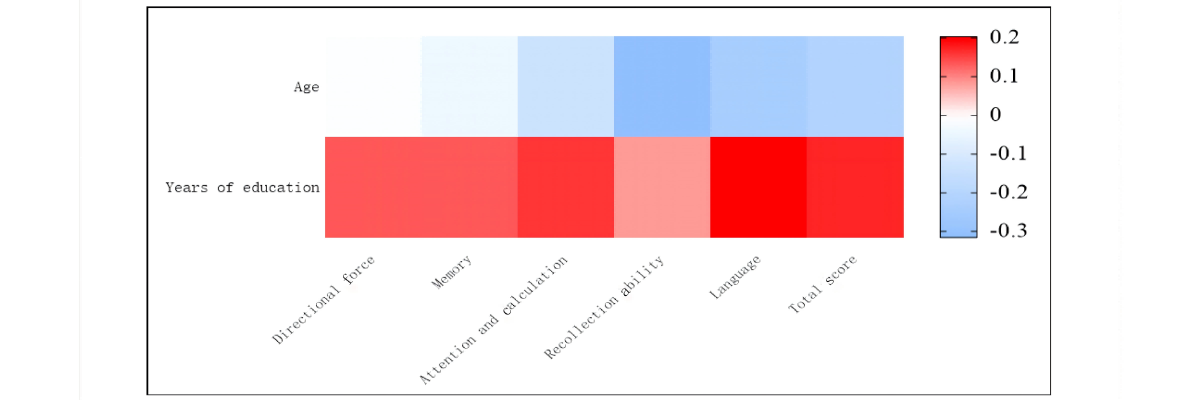
Correlation matrix of MMSE scores with age and education level. The figure shows correlation coefficients (R) on the right; all *P* values were less than .05. MMSE: Mini-Mental State Examination.

Correlation analyses were conducted to explore the relationships between clinical variables and cognitive function. The results indicated that MMSE scores were significantly negatively correlated with age (*r*=–0.32; *P*<.01) and significantly positively correlated with years of education (*r*=0.41; *P*<.01). These findings suggest that older age is associated with lower cognitive function, while higher educational attainment is linked to better cognitive performance following a stroke (Figure 2).

### Cognitive Function by Age Group

**Figure 3:**
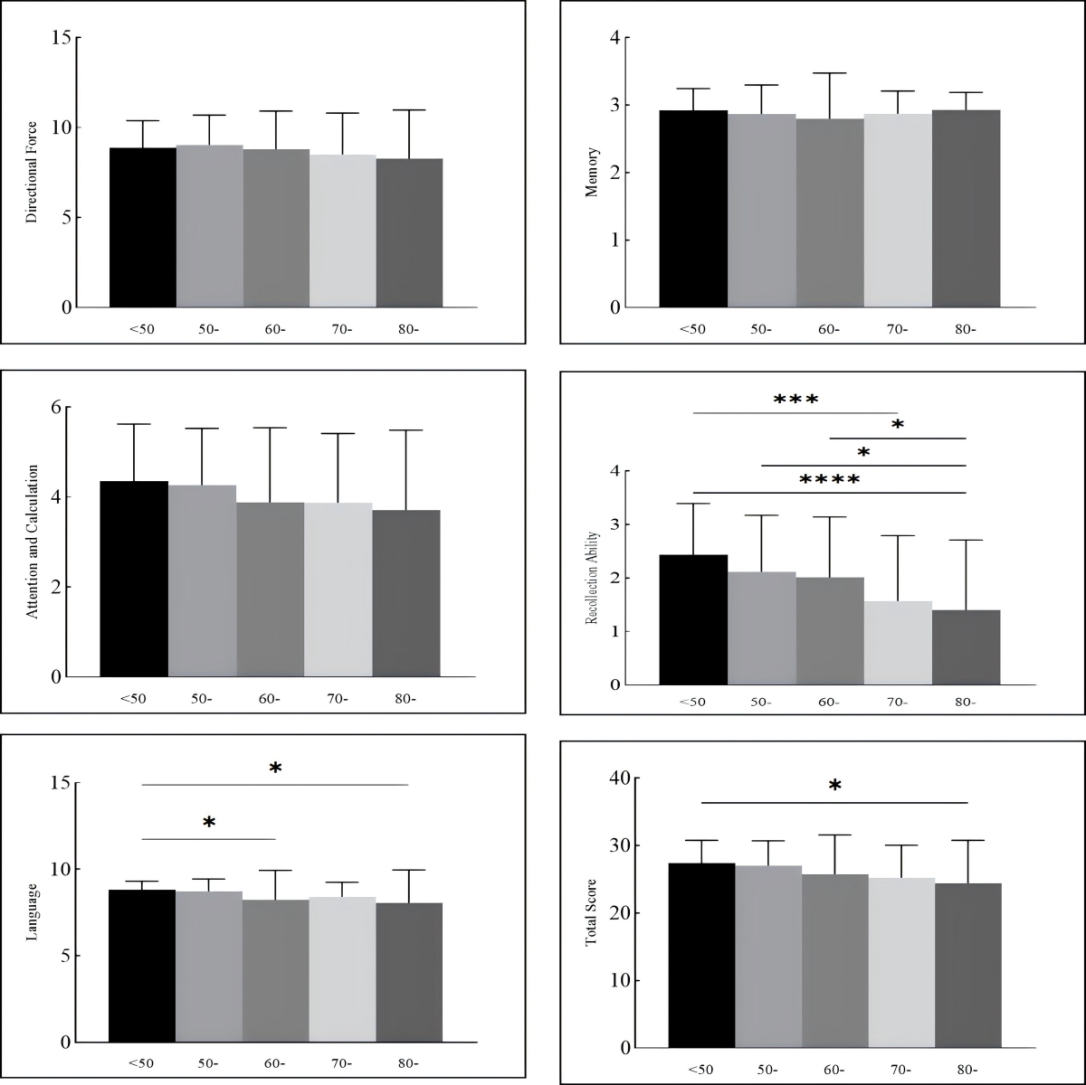
Differences in specific cognitive functions of patients with stroke by age group. *: *P*<.05; **: *P*<.01; ***: *P*<.001. The x-axis represents different age groups: <50, 50-59, 60-69, 70-79, and ≥80 years).

An analysis of cognitive function based on age groups was conducted to assess how aging impacts cognitive outcomes among stroke survivors. The results showed no significant differences in cognitive function in the orientation and memory domains across different age groups (*P*=.49 for memory, *P*=.35 for orientation). However, overall cognitive function and scores in other domains demonstrated a trend of decline with increasing age, particularly among patients older than 60 years (Figure 3). This trend underscores the impact of aging on cognitive function in stroke survivors and highlights the need for age-specific interventions.

### Cognitive Function by Educational Level

**Figure 4:**
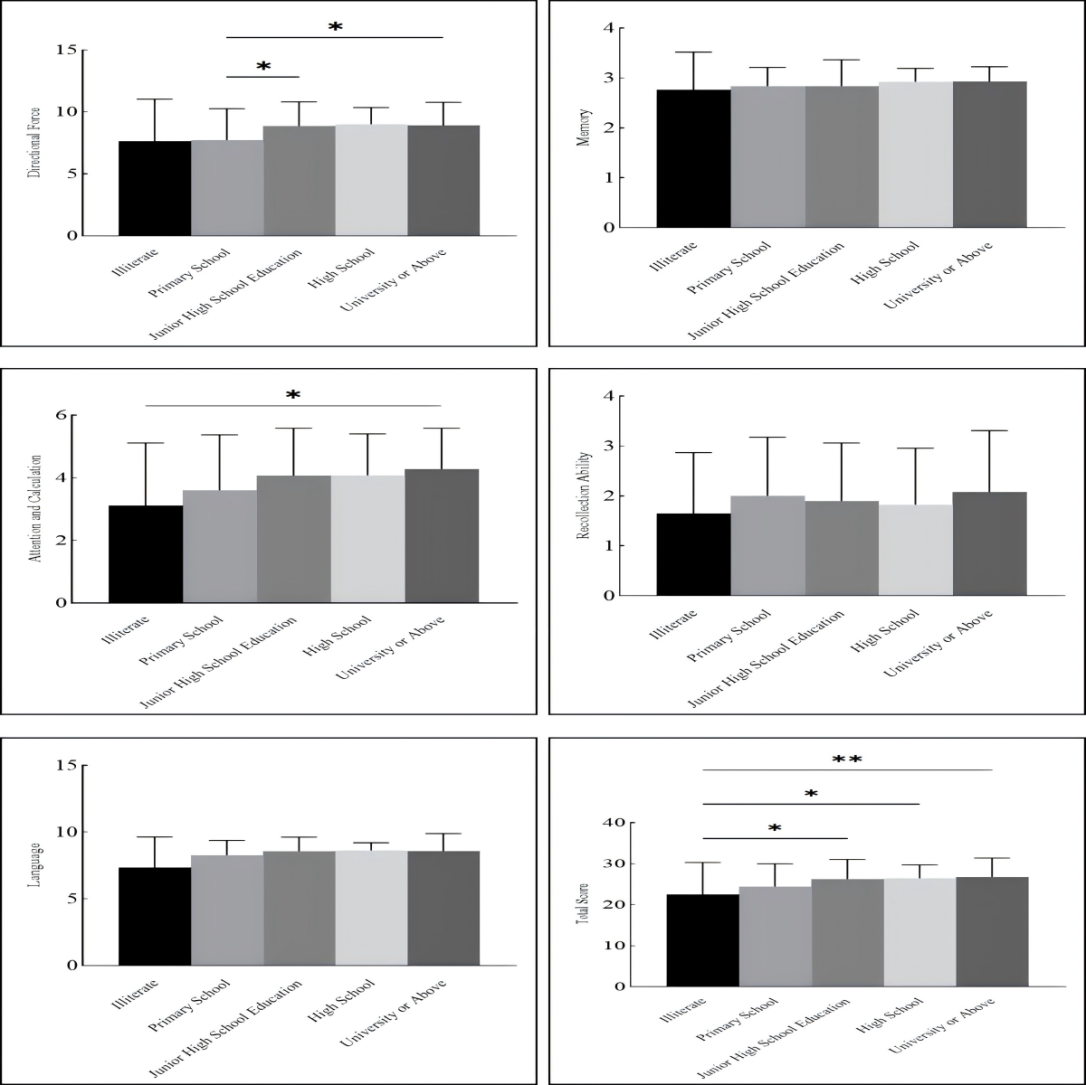
Differences in specific cognitive functions of patients with stroke by educational level. *: *P*<.05; **: *P*<.01; ***: *P*<.001. The x-axis represents different levels of education.

The relationship between educational level and cognitive function was further examined. The analysis revealed a positive association between educational attainment and cognitive function (*r*=0.41; *P*<.01). ANOVA based on educational levels indicated no significant differences in memory (*P*=.36) and recall ability (*P*=.58) among different educational stages. However, overall cognitive function and scores in several domains were higher in patients with higher educational levels. Patients with no formal education had the lowest cognitive scores, while scores improved progressively with higher educational attainment (Figure 4).

### Cognitive Impairment in All Cognitive Domains

The study also assessed cognitive impairment across all cognitive domains as measured by the MMSE. Patients with stroke identified as cognitively impaired (n=51) had significantly lower scores across all MMSE domains compared to those without cognitive impairment (n=254), except for recall ability, which did not show a statistically significant difference (*P*=.16). This finding suggests that stroke impacts multiple cognitive domains, not limited to memory alone, indicating a broad spectrum of cognitive deficits in this population (Table 3).

However, we recognize the potential redundancy in comparing groups defined by MMSE scores using the same MMSE measure. Therefore, future research should consider using additional cognitive assessments that can provide more specific insights into different cognitive domains and better distinguish between different levels of cognitive impairment.

**Table 3:** Comparison of cognitive profiles between cognitive impairment groups.

	No cognitive damage (n=254), median (IQR)	Cognitive damage (n=51), median (IQR)	*z* score	*P* value
Age	62 (51-73)	73 (59.5-79.5)	–1.826	.07
Education	9 (9-15)	9 (8.75-12.75)	–0.336	.73
Directional force	10 (9-10)	5.5 (3-7.25)	–0.227	.02
Memory	3 (3-3)	3 (2-3)	–0.732	.02
Attention and calculation	5 (4-5)	1 (0-3)	–0.295	.005
Recollection ability	3 (2-3)	0 (0-1)	–0.024	.16
Language skills	9 (9-9)	8 (6-9)	–0.702	<.001
Total score	28 (26-30)	18 (13-22)	–0.014	.003

## Discussion

### Principal Findings

Stroke is a neurological syndrome characterized by either localized or generalized cerebral deficits resulting from acute disruptions in cerebral circulation. It primarily includes 2 major categories—ischemic and hemorrhagic strokes. Ischemic strokes occur due to cerebral infarction, while hemorrhagic strokes involve cerebral hemorrhage and subarachnoid hemorrhage. Cognitive function encompasses various mental activities of the brain, such as perception, memory, language, and executive function. Cognitive impairment, which often manifests as an important feature of dementia, can progress over time from mild cognitive impairment to severe cognitive dysfunction and dementia [16].

Ischemic strokes not only lead to neurological deficits, such as limb numbness, weakness, and swallowing difficulties, but also frequently result in cognitive impairments, including memory loss, speech difficulties, diminished attention, visuospatial impairment, and executive dysfunction—collectively referred to as PSCI. Cognitive impairment can manifest at any time following a stroke and is often insidious in onset, heterogeneous in presentation, and easily overlooked. Without early detection and intervention, cognitive impairment can progressively worsen, potentially leading to dementia, thereby significantly affecting the quality of life for patients with stroke and their families. Early identification and treatment of cognitive impairment are, therefore, critical to prevent or delay the onset and progression of dementia in patients with stroke. Consequently, PSCI has attracted increasing attention from stroke researchers worldwide for its role in guiding treatment and rehabilitation strategies aimed at improving patient prognosis.

The reported incidence of PSCI varies widely across studies, potentially due to differences in geographical location, ethnicity, timing of assessments, assessment methods, and diagnostic criteria. For instance, a cross-sectional community-based study in China involving 599 patients with stroke assessed with the Montreal Cognitive Assessment (MoCA) and MMSE scales reported a PSCI prevalence of 80.97%, with 48.91% of cases classified as nondementia cognitive impairment and 32.05% as poststroke dementia [17]. In contrast, another study observed that the incidence of PSCI at 3 months post stroke in China ranged from 18% to 41.8% [18]. Furthermore, a multicenter prospective cohort study in Korea, assessing the cognitive function of 353 patients with stroke at a 3-month poststroke follow-up, found an incidence of PSCI as high as 62.6%, with 49.9% representing nondementia cognitive impairment [19]. In a recent study, it was found that multifaceted assessments could significantly influence functional outcomes in stroke survivors, highlighting the importance of comprehensive evaluations in this population [20]. To explore long-term cognitive changes, a 1-year follow-up study in Singapore involving 252 patients with stroke found that 44% experienced cognitive decline at 6 months post stroke, with the incidence decreasing to 34% at 1 year [21]. A Norwegian study reported that 37.5% and 19.6% of patients exhibited mild cognitive impairment and dementia, respectively, 1 year after their first stroke [22].

In this study, we found that 16.7% of the 305 patients with stroke assessed with the MMSE exhibited cognitive dysfunction. This prevalence is lower than that reported in many other studies, potentially due to differences in the timing of the assessment post stroke and the study’s focus on patients during their hospital stay. The timing of cognitive assessments post stroke is critical, as cognitive function can fluctuate during the acute and subacute phases of recovery [23]. Moreover, Shin et al [24] emphasized the effect of cognitive reserve on recovery trajectories, suggesting that those with a higher cognitive reserve may experience better outcomes post stroke. Future research should consider multiple assessments over time to capture the dynamic nature of cognitive recovery and decline.

Despite the significant cognitive damage observed in patients with stroke, this study did not find a difference in recollection ability between groups. This lack of difference may suggest that the mechanisms of memory processing in patients post stroke are more uniform than previously thought, potentially influenced by the nature of the brain injuries involved. Furthermore, the presence of a substantial numerical difference between the cognitive damaged and undamaged groups could impact the validity of comparisons, as the larger cognitive impairments might obscure nuanced differences in recollection abilities. This highlights the need for a more refined assessment approach that could differentiate between various types of cognitive deficits more effectively.

This study did not find significant differences in cognitive function between male and female patients with stroke, consistent with some previous research suggesting that gender may not significantly influence PSCI [25]. However, other studies have reported a higher incidence of cognitive impairment in men than in women [26]. Furthermore, one study discusses gender differences in mortality and long-term functional outcomes post stroke, suggesting that these factors may also play a role in cognitive recovery and impairment, thereby warranting further investigation into how gender influences PSCI outcomes [27]. Age is widely recognized as a risk factor for both stroke and cognitive impairment. Many studies, including this one, have demonstrated that advanced age is associated with a higher risk of PSCI and that cognitive function tends to decline with increasing age [28]. Our findings also indicate that cognitive function significantly decreases in patients with stroke older than 60 years, with those aged 60-69 years exhibiting more pronounced cognitive impairment compared to those aged 50-59 years. This pattern suggests that cognitive impairment may become more evident in patients with stroke as they age, possibly due to the combined effects of vascular damage from the stroke and age-related neurodegenerative changes.

Typically, cognitive abilities peak around the age of 35 years, begin to decline gradually thereafter, and experience a more rapid decline around the age of 60 years [29]. In patients with stroke, cognitive function may further decline after 60 years of age due to brain atrophy, white matter degeneration, and the location of the stroke lesions. PSCI results from a complex interplay of vascular risk factors and neurodegenerative processes. High age, low education level, predominance of manual labor, diabetes, long-term alcohol consumption, recurrent strokes, lesions in key brain areas, severe neurological deficits, and low ability to perform daily living activities have all been identified as independent risk factors for PSCI. To reduce the incidence of PSCI, it is crucial to improve public education, promote healthier lifestyles (including alcohol cessation), manage diabetes effectively, implement primary stroke prevention strategies, and provide comprehensive poststroke rehabilitation. These strategies are vital to mitigate the risk of cognitive impairment following a stroke [16,30].

Education level is closely linked to cognitive outcomes post stroke. Patients with lower educational attainment are more likely to develop cognitive dysfunction following a stroke [31]. Studies using the MoCA have shown that patients with lower education levels exhibit poorer overall cognitive function [32]. This study similarly found that patients with fewer years of education were more prone to cognitive dysfunction after a stroke, while higher education appeared to be a protective factor. The protective effect of higher education may be due to greater cognitive reserve, which helps delay the onset of cognitive impairment. Patients with higher education levels may have stronger baseline cognitive function before the stroke, enabling better cognitive performance post stroke despite comparable levels of stroke-induced brain injury. Education is also associated with synaptic plasticity; higher education levels promote neurogenesis and synaptogenesis. Individuals with higher education may be more capable of recruiting alternative neural networks to maintain cognitive function. Additionally, patients with higher education levels often have higher socioeconomic status, healthier lifestyles, better adherence to vascular risk management, and greater access to medical resources following a stroke. Therefore, clinicians should be vigilant for PSCI in older patients with stroke with low education levels, as they are at higher risk.

The results of this study also indicate significant declines across multiple cognitive domains—such as orientation, memory, attention, calculation, recall, and language skills—in patients with stroke with cognitive impairment. These findings suggest that cognitive impairment following a stroke is often widespread, affecting various aspects of cognitive function. Patients with nondementia cognitive impairment post stroke may exhibit deficits in attention, executive function, memory, and language, even if they do not meet the diagnostic criteria for dementia. This population has a high rate of progression to dementia [33]. Therefore, it is crucial to identify this group early and provide targeted cognitive interventions to promote recovery and prevent further cognitive decline.

Recent advances in neuroimaging and neuropsychology have shed light on the intricate mechanisms underlying PSCI. Studies using advanced imaging techniques, such as diffusion tensor imaging and functional MRI, have revealed that cognitive impairment in patients with stroke is not merely the result of localized brain damage but also involves widespread network disruptions that affect global brain connectivity [34]. These disruptions can impair cognitive domains such as attention, executive function, and memory, which are crucial for daily functioning. This network-based perspective on PSCI aligns with the growing recognition that cognitive recovery depends on the brain’s ability to reorganize and form new connections, a process known as neuroplasticity [35]. The extent and pattern of network disruption, as well as the individual’s capacity for neuroplasticity, may partly explain the variability in cognitive outcomes observed in our study and others. Future studies should incorporate multimodal imaging approaches to further elucidate how stroke-induced changes in brain networks contribute to specific cognitive deficits and recovery patterns. Such research could pave the way for more personalized rehabilitation strategies that target the unique network dysfunctions of individual patients.

Moreover, emerging evidence suggests that the gut-brain axis may play a significant role in cognitive outcomes following a stroke. Recent studies have highlighted that alterations in gut microbiota composition—collectively referred to as dysbiosis—can influence neuroinflammation and cognitive function [36]. For instance, changes in gut microbiota can modulate the production of short-chain fatty acids and neurotransmitters, which in turn affect brain function and cognitive performance [37]. Given the high prevalence of gastrointestinal comorbidities and the frequent use of antibiotics and other medications in patients with stroke, it is plausible that poststroke dysbiosis could exacerbate cognitive decline. Incorporating assessments of gut microbiota composition and function into future PSCI research could offer novel insights into the biological underpinnings of cognitive impairment and recovery after a stroke. This holistic approach might also reveal new therapeutic targets, such as probiotics or dietary interventions, to improve cognitive outcomes in stroke survivors.

One important aspect not fully addressed in this study is the differentiation between stroke stages—acute, subacute, and chronic—which could have provided more nuanced insights into the progression of PSCI. The exclusion criteria did not specifically distinguish between these stages, and while the study focused on patients during hospital admission, the stage of stroke might significantly influence cognitive outcomes. Acute-stage patients, for example, may exhibit different cognitive impairments compared to those in the subacute or chronic stages. Future research should aim to differentiate between these stages to better understand how timing post stroke affects cognitive function and rehabilitation outcomes.

Additionally, although the MMSE was used as the cognitive assessment tool, it is recognized that the MoCA could provide a more sensitive measure for detecting mild cognitive impairment, especially in patients with vascular origins of cognitive dysfunction. While the MMSE is widely used in clinical practice, it may lack the specificity needed for detecting subtle cognitive deficits that the MoCA could capture. A future study incorporating the MoCA alongside the MMSE would likely yield a more comprehensive understanding of cognitive impairment in stroke survivors, allowing for a broader spectrum of cognitive deficits to be assessed.

In conclusion, cognitive impairment in patients with stroke is primarily manifested as a decline across multiple cognitive domains, with aging being a significant risk factor. To prevent further cognitive deterioration at a younger age, it is essential to explore the mechanisms underlying age-related cognitive impairment in patients with stroke. Such exploration will lay a theoretical foundation for developing effective prevention strategies for cognitive impairment in this population.

### Limitations

This study has several limitations that should be considered when interpreting the findings. First, the data were collected from patients at a single hospital, which may limit the generalizability of the results to a broader population. Future studies should involve multicenter collaborations to ensure a more diverse and representative sample. Second, the relatively small sample size may introduce data bias, and future research should aim for larger sample sizes to enhance the reliability of the results. Third, the cross-sectional design restricts the ability to draw causal inferences about the relationship between stroke and cognitive impairment. Longitudinal studies with extended follow-up periods could provide deeper insights into the progression of cognitive impairment over time.

Additionally, this study did not account for stroke severity, a critical factor that can heavily influence cognitive outcomes. Excluding patients with severe strokes may have led to an underestimation of cognitive impairment rates, as more severe cases were not represented in the analysis. Future research should incorporate a broader range of stroke severities to better capture the spectrum of cognitive impairments. The lack of differentiation between stroke stages (acute, subacute, or chronic) also limits the understanding of how timing post stroke impacts cognitive function, as stroke stage may significantly affect recovery and impairment levels. Finally, while the MMSE was used to assess cognitive function, this tool may not have been sufficiently sensitive to detect milder forms of cognitive impairment. The inclusion of more specific measures, such as the MoCA, in future research would help provide a more thorough evaluation of cognitive deficits, especially those of a subtler nature.

### Conclusions

This study emphasizes the significant roles of age and education in related poststroke cognitive outcomes and underscores the value of identifying these factors for early prevention and intervention strategies. By analyzing the factors associated with cognitive dysfunction after stroke, the findings provide crucial insights that can guide treatment and rehabilitation approaches, ultimately improving patient prognosis. Future research should build on these results by incorporating a broader range of variables and using more advanced research designs to deepen our understanding of PSCI and enhance clinical practice.

## Appendix

Multimedia Appendix 1 STROBE (Strengthening the Reporting of Observational Studies in Epidemiology) checklist on cross-sectional studies.

## Abbreviations

ANCOVA: analysis of covariance
CT: computed tomography
MMSE: Mini-Mental State Examination
MoCA: Montreal Cognitive Assessment
MRI: magnetic resonance imaging
PSCI: poststroke cognitive impairment
STROBE: Strengthening the Reporting of Observational Studies in Epidemiology

## Data Availability

The datasets generated and analyzed during this study are available from the corresponding author on reasonable request.
